# Maneuvers for the treatment of benign positional paroxysmal vertigo: a systematic review

**DOI:** 10.1016/S1808-8694(15)30046-X

**Published:** 2015-10-19

**Authors:** Lázaro Juliano Teixeira, João Natel Pollonio Machado

**Affiliations:** a Neurologic Physical Therapy Specialist – Professor of Physical Therapy applied to Neurology – University of Vale do Itajaí, Univali, Itajaí, SC. Physical Therapist of the Balneário Camboriú City Hall, Balneário Camboriú, SC.; b MSc in Medical Sciences – Federal University of Santa Catarina, Specialization and Medical Residence in Neurology – Federal University of Paraná. PhD student in Neurology – University of São Paulo - Ribeirão Preto. MD. Neurology Professor – University Regional de Blumenau - FURB, Blumenau, SC.

**Keywords:** Vestibular Diseases, Vertigo, Rehabilitation, Physical Therapy, Meta-analysis, Literature Review

## Abstract

Benign Paroxysmal Positional Vertigo (BPPV) is one of the most frequent diseases of the vestibular system and it is characterized by episodes of recurrent vertigo triggered by head movements or position changes. There are several approaches for treatment, but efficacy is still being discussed.

**Aim:**

To asses the effectiveness of the specific maneuvers available to the treatment of BPPV.

**Methodology:**

An electronic search at the main databases, including MEDLINE, LILACS, PEDro, Cochrane Collaborations Database was performed, and we selected only randomized clinical trials studying adults with diagnosis of BPPV confirmed by the Dix-Hallpike test. The trials should have included physical maneuvers such as Epley and Semont. The main outcome was Dix-Hallpike negative test and the changes to subjective complaints. The trials were assessed using Jadad's scale and only studies with quality scores equal or above 3 were pooled on a meta-analyses to assess their effectiveness.

**Results:**

We found five controlled clinical trials phase I comparing the Epley's maneuver with controls or placebo. The metaanalysis showed positive evidence of Epley's maneuver to the posterior semicircular canal (effect size = 0.11 [CI 95% 0.05, 0.26] of objective improvement [Dix-Halpike] within one week, 0.24 [CI 95% 0.13, 0.45] within one month and 0.16 [CI 95% 0.08, 0.33] of improvement reported by the patients within one week. There are no studies about the efficacy of Semont's maneuver.

**Conclusion:**

There is scientific evidence showing good efficacy of Epley's maneuver in the treatment.

## INTRODUCTION

Benign paroxysmal positional vertigo (BPPV) is one of the most frequent vestibular disorders^1^, ^2^. It is clinically characterized by recurrent vertigo spells, usually triggered by certain head movements or patient's change in posture^3^.

Diagnosis is clinical. The interview reveals a typical history with short vertigo spells at head movements^4^. Because of its clinical characteristics, patients feel fearsome, and both vertigo as well as triggering head movements might considerably limit their daily activities^5^. Symptoms tend to spontaneously resolve after a few weeks or months. However, some patients experience recurrent symptoms months or even years later, which may vary from short spells to decades of suffering, with short remission spans^5^.

Dix-Hallpike maneuver aids in diagnosis. We have a positive maneuver when it triggers vertigo and nystagmus when the patient changes posture from sitting to laying down with his/her head hanging downwards horizontally, with a 45° head turn towards the tested side^3^, ^5^, ^6^. Rotational nystagmus is typical: four to five second latency and duration of 30 to 40 seconds. As we repeat the maneuver, fatigue ensues, reducing nystagmus intensity until it totally recedes in the third or fourth repetition.

BPPV clinical findings agree with the hypothesis that semicircular canals, with greater incidence on the posterior canal, have floating particles or debris, which are heavier than the circulating endolymph^5^.

Although the exact mechanism by which these debris cause BPPV and nystagmus is still unknown^1^, it is broadly accepted that a canal lithiasis phenomenon be responsible for this condition^6^.

Each free debris point require a different treatment strategy, through maneuvers comprised of head movements, in order to restore normal semicircular canal function and thus eliminate vertigo and positional nystagmus^7^.

This therapy involves head position changes in a series of repetitions, as proposed by Brandt and Daroff^8^, Semont´s releasing maneuver, Epley's canalicular repositioning^1^, ^9^, among others^10^, ^11^, ^12^, ^13^. The main goal of these maneuvers is to take the free debris from the semicircular canal back to the utricle, where they presumable adhere1. Head position exercises attempt to reach central nervous system adaptation and compensation mechanisms, trying symptom recovery.

There are three basic BPPV treatments, each with its own use indication: canal repositioning, releasing exercises and habituation exercises. Efficacy studies state that all three facilitate recovery. We typically use canal repositioning treatment or releasing maneuvers. Habituation exercises are used for milder residual complaints^2^. Some papers have shown little effect of canal repositioning maneuvers as to long lasting symptoms improvement, as well as weak evidence when compared to other therapeutic resources (physical therapy, medical or surgery related) for posterior semicircular canal BPPV, especially due to a lack of good quality clinical studies^6^, ^14^.

Van der Velde^15^ analyzed other conservative and non-pharmacological physical treatments besides repositioning maneuvers. His conclusions are that these maneuvers efficacies are not yet satisfactorily determined.

Herdman and Tusa^2^ Report some controversies regarding canal repositioning maneuvers. They mention some studies which show 85 to 95% of symptoms remission in posterior canal BPPV patients, however those were studies without control groups, and spontaneous recovery could not be ruled out.

Even if we establish that physical therapy resources (exercises and specific maneuvers) are of great value for vertigo treatment^16^, we know that only clinical trials may check their reliability, tolerance, effectiveness and efficacy^17^.

Thus, we justify this review because of the need to group scientific evidence that show an efficacy measure for these maneuvering treatments proposed to treat BPPV, and we enquire: Is physical therapy intervention, through debris releasing maneuvers effective to treat BPPV?

The Goal of this review is to assess releasing maneuvering efficacy in BPPV diagnosed patients.

## STUDY METHODOLOGY

### Population and Sample

This investigation was carried out in the following electronic data bases: LILACS (1982 until August 2004), MEDLINE (January 1966 until August 2004), Cochrane Register of controlled studies (2004/3 issue), PEDro (Physiotherapy Evidence Database (1999 until August 2004). We also carried out new electronic and manual search in the references mentioned by the papers studied, in theme-related electronic sites, national and international journals, and we also used the OVID search engine.

### Search Strategy

The search strategy we used followed the recommendations by Dickersinet al.^18^, Castro et al.^19^, Systematic Reviews Cochrane Manual^20^ and Bickley and Harrison^21^. We used the expressions and combinations described on Table 1.

**Table 1:** Search strategy used in this review

#1 vertig*
#2 dizz*
#3 benign
#4 paroxysmal
#5 (#2 or #3 or #4)
#6 Epley
#7 Semont
#8 canalith*
#9 particle*
#10 position*
#11 (#6 or #7 or #8 or #9 or #10)
#12 clinical trial
#13 randomized controlled trial*
#14 randomized clinical trial*
#15 double blind*
#16 comparative study*
#17 (#12 or #13 or #14 or #15 or #16)
#18 (#5 and #11 and #17)

Inclusion criteria for the studies: randomized, controlled clinical prospective studies, involving individuals older than 18 years with BPPV clinical diagnosis, confirmed by the Dix-Hallpike positional test with classical signs of positional nystagmus. Interventions could have been by specific maneuvers (Epley, Semont, etc.), or positional exercises, habituation, adaptation or compensation, compared to other interventions such as placebo, medication or surgical procedures. Expected outcomes included the patient's functional improvement in their daily lives and negative result in the Dix-Hallpike test. We also considered the following outcomes: vertigo spells frequence and severity and proportion of patients who reported improvements with the intervention. We selected only papers written in Portuguese, English or Spanish.

Exclusion criteria: other labyrinth diseases: Ménière disease, vestibular neuronitis, other peripheral vertigos, other vestibular function disorders, labyrinthitis, labyrinth fistula, labyrinth dysfunction and central origin vertigo. We also excluded those studies in which the primary therapy was related to physical changes in the individual's environment (removing rugs, using lighting or signaling, etc), the use of movement aiding equipment, as well as papers which analyzed other forms of physical therapy intervention such as electrotherapy, electrical stimulation (functional, neuromuscular), transcutaneous electric neurostimulation (TENS).

### Data Collection instruments

Papers were assessed as to methods quality, in a non-blinding way by the first author of this paper. Despite the criticism regarding the assessment of work quality through a scale that primarily measures the quality reported along the analyzed study^22^, we used Jadad's Scale because it is easier^23^. Jadad's scale comprises the answer to five questions: was the study described as being randomized? Was the randomization method adequate? Was the study described as being double blind? Was the masking method properly used? Were losses and withdrawals described? Each positive answer generates 1 point in the scale that varies from 0 to 5 points. 1 and 2 point clinical studies were considered low quality, and 3 to 5 point studies were considered high quality. Data analysis considered only data from 3 to 5 point studies.

Data Treatment: After qualitative analysis, the studies were classified in subcategories according to: 1. Intervention mode (Semont's maneuver, Epley's maneuver, other); 2. Follow up period (assessment made in days, weeks or months) and according to intervention type. Statistical analysis and metanalysis were carried out using the RevMan 4.2 software. All variables were considered dychotomic data, in other words, improvement is equal to negative Dix-Halpike or patient reported total improvement; or shown on quality scales used. For that we used relative risk (RR) and 95% confidence interval through a fixed effect model to interpret the results.

## RESULTS

Some searched listed papers could not be located^24^ and others were not analyzed because they were written in a foreign language which was not part of those listed in the inclusion criteria^25^, ^26^, ^27^. Twenty-nine papers were excluded for different reasons. These studies, their quality assessment results and their exclusion criteria are listed on Table 2.

**Table 2:** Evaluation of the studies as to the Jadad scale.

STUDY (Year, Author)	JADAD	REASON FOR EXCLUSION
Firrisi et al.24	X	Not found
Aso et al.26	X	Original in Japanese, paper not requested
Pampurik et al.27	X	Original in French, paper not requested
Kammerlind et al.42	X	Not peripheral origin vertigo and instability patients, paper not requested
Ganança43	X	This was no scientific paper
1998, Yardley, et al.44	3	Heterogenous sample, there was no Dix-Hallpike confirmation and included other labyrinth diseases such as Ménière.
2000, Moreno, Renaud45	0	Not a randomized study
2001, Sargent et al.46	0	Not a randomized study
2002, Gains e Gains47	0	Retrospective study, not randomized
1993, Herdman et al.48	1	Described as randomized but without a description of the randomizing
1994, Fujino et al.49	1	Not a randomized study
1995, Li50	1	Described as randomized but without a description of the randomizing
1996, Massoud, Ireland33	1	Described as randomized but without a description of the randomizing
1997, Smouha	1	Loss or follow up waivers described
1998, Nuti et al.51	1	Loss or follow up waivers described
1998, Wolf et al.52	1	Described as randomized but without wrongly randomized
1999, Radtke et al.53	1	Loss or follow up waivers described
2000, Nuti et al.54	1	Loss or follow up waivers described
2001, Sherman e Massoud55	1	
2002, Reis et al.56	1	Loss or follow up waivers described
2004, Macias et al.57	1	Described as randomized but without wrongly randomized
2003, Salvinelli et al.58	1	Described as randomized but without a description of the randomizing
1994, Blakley34	2	Randomized and with loss description, however with wrong randomizing
1996, Steenerson e Cronin38	2	Randomized and with loss description, however with wrong randomizing
1999, Cohen e Jerabek59	2	Only randomized and with loss description, however with wrong randomizing
2000, Asawavichianginda, et al.60	2	Only randomized and with loss description, however with wrong randomizing
2001, Varela, et al.35	2	Randomized and with loss description, however with wrong randomizing
2002. Haynes et al.61		Not a randomized study
2003, Califano, et al.62	2	Randomized and with loss description, however with wrong randomizing
2004, Cohen e Kimball63	2	Randomized and with loss description, however with wrong randomizing
2004, Salvinelli, et al.36	2	Randomized and with loss description, however with wrong randomizing
2004, Radtke et al.53	2	Randomized and with loss description, however with wrong randomizing

After the papers were selected according to inclusion criteria and to methodology quality, there were five Epley's maneuver studies left, comparing them to placebos, no treatment or medication^28^, ^29^, ^30^, ^31^, ^32^.

Lynn et al.^28^ compared Epley's maneuver (n=18) with placebo (n=15) without previous medication or vibration. Medication was allowed after the maneuver, besides recommending the patients to keep their heads up and wearing a neck collar for 48 hours, avoid neck movements and avoid sleeping over the affected side for one week. The patients were reassessed one month after the Dix-Hallpike maneuver and through their personal journals. The test became negative in 88.9% of the maneuver group participants and in 26.7% in the placebo group (p=0.001). Improvement was reported in 61.1% in the treated group and in 20% in the placebo group (p=0.0329).

In a study by Froehling et al.^31^ Epley's maneuver was changed from the original format only as to not using mastoid vibration. The treated group (n=24) was compared to the placebo maneuver group (n=26), performed with the patient laying down over the affected side for 5 minutes. All 50 patients wore the neck collar on the first two nights and were asked not to sleep over the affected side for 5 days, and avoid head movements for one week. Dix-Hallpike^31^ testing re-assessments were carried out after one and two weeks and showed significant differences favoring the maneuver (67% vs 38%, p=0.046).

Angeli et al.^32^ developed a study with 47 senior citizens. The patients were randomly distributed in two groups and Epley's maneuver with mastoid vibration in the experimental group (n=28), there was only one control group (n=19). Post-maneuver recommendations were given, such as to avoid vertigo-provoking movements, avoid sleeping with the head high for 48 hours and, if necessary, use anti-vertigo drugs. A neck collar was used during this period. Reassessment was carried out in one month and the treated group enjoyed 64% of improvement, while the control group had 5.26% (p<0.001)32.

Similarly, Yimtae et al.^30^ studied adding Epley's maneuver to a group with medication and compared it to a group using medication alone (cinnarizin). No other recommendation was given after Epley's maneuver, not even the neck collar. No mastoid vibration was used. The groups were compared after one, two, three and four weeks, with results favoring the maneuver group, specially after one week (75.9% vs. 48.2%, respectively, p = 0.03), reducing from then on until there was no significant difference after one month (96% vs 90%, respectively, p = 0.336). However, patients who underwent Epley's maneuver, besides improving faster, used less anti-vertigo drugs (p=0.001)^30^.

Sridhar et al.^29^ compared 20 patients who underwent Epley's maneuver to 20 control patients who used placebo only. Here, neither mastoid vibration nor medication was used as well, but only sleeping tips such as to elevate bead head for 48 hours. Both groups were followed for one year, with one and four weeks, three, six, nine and twelve months’ reassessment. After one week all patients were Dix-Hallpike test negative, compared to 30% of the control group (p < 0.001). This difference was maintained until the study end, when 95% of the Epley's maneuver patients were described as cured, compared to 25% in the placebo-controlled group (p < 0.001).

## METANALYSIS

**Figure 1:**
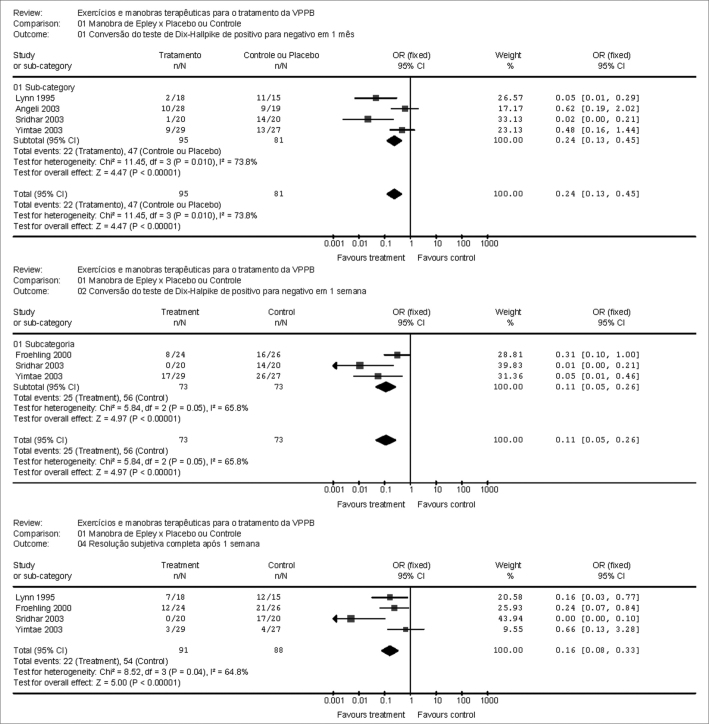
Metanalysis graphs

The filtered papers were then grouped according to follow up time and type of clinical outcome. Thus, it was possible to group them according to: Dix-Hallpike test cure one week after Epley's maneuver; and patient reported subjective improvement one week after the same maneuver. Results are depicted on Figure 1 graphs.

## DISCUSSION

All the studies selected for metanalysis are classified as phase I clinical trials, with small sample sizes and most of them with one month follow up. There is a lack of phases II, III and IV clinical trials, and even multicentric studies, made up of large samples (over 100 individuals) and with longer follow up (over 1 year). There are very few publications either describing or proposing interventions for anterior and horizontal semi-circular canal dysfunctions, and the few studies found were not adequate according to the adopted criteria or Jadad's scale.

We did not find any study on the Semont's maneuver efficacy of enough methodological quality to meet the criteria of this review. A possible explanation is that we did not use a database with mainly European papers, for instance EMBASE. Hilton and Pinder^14^ surveyed this database and obtained results similar to ours, not adding any study from EMBASE. Therefore, we see a broad research field about Semont's maneuver efficacy.

As to the studies found that met the minimum quality requirements to be grouped in our review, we see that only Epley's maneuver was deeply investigated. Notwithstanding, there still remain details and variations as to that author's originally described technique, curiously obtaining similar results. Lynn et al.^28^ kept Epley's maneuver as initially described, although without mastoid vibration or previous medication and allowed post-maneuver use of medication. Froehling et al.^31^ did not use mastoid vibration.

Yimtae et al.^30^ modified body position when carrying out the maneuver, allowing the patient to roll over, belly down after belly up, making a 180° head angle, besides using medication after the maneuver, according to patient needs. Angeli et al.^32^, worked with senior citizens, and they limited neck extension during the maneuver and used mastoid vibration. Sridhar et al.^29^ maintained the original maneuver, but did not use mastoid vibration.

Post maneuver instructions also varied among the papers. In two papers the authors advised patients to wear neck collar after the maneuver for 48 hours^28^, ^32^. Such recommendation was not given in three other studies^29^, ^30^, ^31^.

In three studies, patients were advised to follow restrictive behavior in the first days, such as: sleep while sitting for 48 horas^28^, ^29^, ^31^, avoid provoking movements^28^, ^31^, ^32^, and avoid sleeping over the affected side for one week^28^ or five days^31^. Yimtae et al.^30^ was the only study that made no restrictions or recommendations. This issue was specifically analyzed by Massoud and Ireland^33^ and they did no find statistically significant differences among groups advised for and against restrictions after maneuver. Thus, the use of mastoid vibration, mild maneuver changes, neck movement restrictions with neck collars and advice to restrict movements or not to lay down over the affected side after the maneuver, don’t seem to influence therapeutic results, because the results of studies with and without such limitations are similar, as shown by the metanalysis, in other words, almost all studies represented by horizontal lines are to the left of the vertical line, showing a benefit with the proposed treatment mode. Besides variations on maneuvering techniques, there also are variations as to group follow up. We were able to group trial outcomes with one week and one month follow ups, objectively analyzing it through a negative Dix –Hallpike test. We were also able to group patient objective improvement results one week after the maneuver. Only one year long follow ups of BPPV patients has been considered important in the studies.

Sridhar et al.^29^ made the only trial that followed the sample for one year. All others followed their samples for one month at most, and Froehling et al.^31^ had only a 2 week follow up. Sridhar et al. showed that the maneuver positive result was kept for one year, besides presenting a considerably lower level of recurrence (10% in the Epley's maneuver group vs 90% with placebo).

The effect magnitude of the different follow up was of 0.11 [IC 95% 0.05, 0.26] of objective improvement (Dix-Halpike) one week after, 0.24 [IC 95% 0.13, 0.45] one month after and 0.16 [IC 95% 0.08, 0.33] of patient reported improvement after one week, and this leads us to notice a good improvement, for both physical exam and patient complaints in the first week, however less important in the first month. Notwithstanding, we lack enough scientific evidence to conclude that such benefit would last longer.

Despite this lack of stronger evidence towards BPPV management, there are proposals, which may not be uniformly proved by properly designed clinical studies, but still being part of an arsenal of options useful to treat these patients. These proposals varies from no treatment at all^34^, all the way up to a combination of the numerous existing maneuvers and exercises^32^, ^35^, ^36^.

In the elderly population, these maneuvers might have to be modified to gentler and slower movements with neck rotation limitation. When these maneuvers don’t work or when they may not be properly performed due to limitations caused by old age co-morbidities, additional modalities may be proposed as vestibular habituation exercises^32^.

Other treatment variations may crop up. In a recent prospective study with 247 patients, Gordon et al. reported that trauma resulting BPPV cases are much less frequent than idiopathic origin BPPV cases (8.5% in this study are trauma-related), they are, however, more difficult to treat, requiring repeated treatment sessions to completely resolve the symptoms (77% vs 14%, respectively, p<0.001), besides having greater recurrence trends (57% vs 19%, respectively, p<0.004)^37^.

Steenerson contributes showing in his study the physical therapists usual modus operandi, with many weekly sessions for patients treated by canal repositioning maneuvers and for patients treated by vestibular rehabilitation. With his work it is possible to highlight the importance of continuous follow up and interventions to treat BPPV, in order to facilitate patients’ understanding in carrying out the exercises, and allowing the patients to learn about self treatment – important in recurring cases^38^.

There were very few studies describing BPPV treatment adverse effects. Yimtae et al.^30^ mentions the following as Epley's maneuver adverse effects: fainting, sweating, skin paleness and hypotension that may be caused by limbic system activation due to a repetition of the vertigo inducing procedure. These symptoms were found in 6.9% of the patients who took part in this study. Froehling et al.^31^ mentions vomits during treatment and difficulties in tolerating the maneuver due to neck problems.

The literature describes other systematic BPPV treatment reviews^14^, ^15^, ^39^. Their findings, when compared among themselves and present review results, show some variations. Maher^40^ states that this can be expected, showing search strategies, the data bases used, the methodological quality measuring method for the studies and results grouping method used as modifying factors even at review conclusion. The most complete review was reformulated last year^14^ and sought papers in MEDLINE (1966-2004), EMBASE (1974-2004) and in Cochrane clinical studies register (2004). 296 papers were initially identified, 19 were selected and 15 were analyzed. These figures are similar to ours. Of these, 3 papers were grouped^28^, ^30^, ^31^ making up a total of 144 patients. Our search found one paper more than this aforementioned review, one of the methodologically best carried out and with the larger follow up^29^.

In the above review, studies were described as being of low methodological quality^14^. The studies most important limitations were allocation hiding and evaluator masking, and such factor was also observed with the use of Jadad's scale in this paper. The authors concluded that there is some evidence that Epley's maneuver is safe and effective in treating posterior semicircular canal BPPV; however, they did not find evidences that this maneuver promotes long term symptom resolution. This conclusion could have been a little different if Sridhar et al’.^29^ trial had been considered.

Hilton and Pinder also concluded that there are no evidences of maneuver comparison to other therapeutic, medical or surgical resources to treat posterior canal BPPV, and we agree with such data^14^.

## CONCLUSION

Kinesiotherapy, through Epley's maneuver is efficient for BPPV treatment when compared to placebo and/or drug therapy alone and/or without intervention. Notwithstanding, randomized clinical assays that allowed us these conclusions are phase I, with small samples and short follow up, thus limiting this finding strength.

We did not find evidences about the efficacy of the Semont's maneuver in this review, thus it is not possible to corroborate or refute Semont's maneuver efficacy for BPPV treatment.

We also did not see methodological relevant papers that described or proposed a proper handling of anterior and horizontal canal dysfunction.

We may state that, so far, the use of mastoid vibration, subtle changes in the maneuver movements, restriction of neck movements through the use of a neck collar, movement limitation recommendations, not to lie down on the affected side after the maneuver don’t not seem to influence therapeutic results.

## FINAL RECOMMENDATIONS

Because of the lack of controlled and randomized clinical trials using the Semont's maneuver, we see a broad research field open as far as this maneuver efficacy is concerned.

We suggest phase II, III and IV clinical trials, or even multicentric trials regarding BPPV management not only for the posterior canal but also for the other ones. The studies should be properly designed; especially as proper randomizing methods are concerned, reducing errors and masking, at least the investigator as to the therapy method used.

We also recommend that the study should not involve only otolaryngologists and neurologists, but all the other health care professionals involved in BPPV patient treatment such as general practitioners^1^, emergency physicians^4^, psychiatrists^41^ and physical therapists^2^, we must be attentive to identify BPPV early on, know its main differential diagnosis and its treatment.

Dix-Hallpike maneuver use in the usual testing of vertigo complaining patients may allow the immediate execution of a simple, fast, easy and low cost method, besides having the support of a reasonable amount of scientific evidence.
